# Beyond Human Error: Building Intelligent Resilience for Medication Safety in the ICU

**DOI:** 10.3390/healthcare14050619

**Published:** 2026-02-28

**Authors:** Sung Min Yun, André van Zundert

**Affiliations:** 1 Department of Anaesthesia and Perioperative Medicine, Royal Brisbane and Women’s Hospital, Brisbane QLD 4006, Australia; ysm9030@gmail.com; 2 Royal Brisbane Clinical Unit, Faculty of Medicine, The University of Queensland, Brisbane QLD 4029, Australia

**Keywords:** medication errors, intensive care units, artificial intelligence, predictive analytics, generative AI, implementation science

## Abstract

**Highlights:**

**What are the main findings?**

- Traditional voluntary reporting systems in the ICU miss approximately 98% of medication errors compared to active observation, creating a dangerous surveillance gap.
- We propose a five-layer Intelligent Safety Stack that integrates standardised data, intelligent surveillance, signal optimisation, generative AI, and physical engineering controls to actively detect and intercept errors beyond the limits of human vigilance.

**What are the implications of the main findings?**

- Safety performance should move beyond raw error counts to include rescue rates—the proportion of risks successfully intercepted before patient harm.
- Sustainable safety involves a sociotechnical strategy that addresses implementation barriers such as alert fatigue and data fragmentation rather than relying on adding more digital tools to an already complex workflow.

**Abstract:**

**Background/Objectives:** Medication errors (MEs) in intensive care units (ICUs) remain a persistent threat to patient safety. A significant surveillance gap exists where traditional voluntary reporting detects as few as 0.02 MEs per patient-day, leaving approximately 98% of errors invisible to standard audits. This review critically examines how artificial intelligence (AI) and implementation science can bridge this gap through a proposed five-layer Intelligent Safety Stack. **Methods:** We conducted a critical narrative review of the peer-reviewed literature published between 2000 and 2025, synthesising evidence across medication safety, predictive analytics, generative AI, engineering controls, and sociotechnical frameworks. **Results:** Reported ME incidence varies widely (1.32% to 31.7%) due to the profound methodological heterogeneity. To achieve sustainable safety, we propose a five-layer framework: (1) Standardised Ontology (e.g., NCC MERP) to establish ground-truth data; (2) Intelligent Surveillance to identify and monitor high-risk patients; (3) Signal Optimisation to filter noise and reduce alert fatigue; (4) Generative Stewardship to automate reconciliation at transitions of care; and (5) Engineering Controls (smart pump interoperability and NRFit™), which have been shown to reduce administration errors by up to 54.8%. **Conclusions:** Isolated error counting is insufficient. Sustainable medication safety in the ICU involves a sociotechnical fusion of the Intelligent Safety Stack with success measured by rescue rates rather than error prevalence alone.

## 1. Introduction

Medication errors (MEs) in intensive care units (ICUs) remain a persistent threat, compounded by patients’ extreme physiological complexity [1] and high rates of polypharmacy [2]. Consequently, the ICU’s cognitively demanding environment and complex multidisciplinary workflows frequently compromise safety protocols [3,4].

Although digital tools such as clinical decision support (CDS) systems were implemented to manage this complexity, they inadvertently generated new vulnerabilities. Chief among these is alert fatigue, wherein a high volume of low-value notifications drives clinicians to override the majority of warnings [5].

Addressing these modern vulnerabilities is hindered by a profound surveillance gap. A nearly 50-fold discrepancy exists between active observation and passive reporting [6] and reported ME prevalence varies wildly across studies due to heterogeneous definitions and detection methods [7,8]. To navigate this lack of standardised metrics, it is essential to establish a strict conceptual separation: error detection (identifying a process failure), error interception (halting the error before it reaches the patient), and harm prevention (reducing adverse patient outcomes).

The recent literature pivots toward AI and implementation science to bridge these gaps. Machine learning (ML) tools now enable proactive risk identification [9], while engineering controls such as smart pump interoperability and route-specific connectors enhance physical safety [10,11]. However, because technology alone is insufficient, frameworks like the Consolidated Framework for Implementation Research (CFIR) are increasingly recognised as vital tools to identify sociotechnical barriers [12].

This review synthesises evidence from 2000 to 2025 to move beyond descriptive epidemiology and examine how sustainable medication safety can be achieved in the ICU. We propose a five-layer Intelligent Safety Stack that integrates data architecture, intelligent surveillance, signal optimisation, generative stewardship, and physical engineering controls. Importantly, we frame these interventions through an implementation science lens to ensure these technologies are seamlessly integrated into real-world ICU workflows.

## 2. Methods

### 2.1. Review Design

We conducted a critical narrative review to integrate heterogeneous evidence streams spanning medication safety research, ML validation studies, implementation science frameworks, and engineering-based safety interventions. While recent systematic and scoping reviews have rigorously mapped isolated domains such as the descriptive epidemiology of MEs or the isolated efficacy of AI alerting tools, these structured methodologies are inherently bounded by strict criteria that frequently preclude cross-disciplinary integration. A narrative review was deliberately selected to bridge these gaps. Rather than merely aggregating data on individual technological tools, this interpretive approach enables the conceptual synthesis of disparate scientific streams (engineering, computer science, sociology, and clinical pharmacology) into a novel, cohesive operational framework: the Intelligent Safety Stack. This design is uniquely appropriate for examining complex sociotechnical systems where the interaction between human workflow and technology is the primary focus, and where traditional randomised control trial data remain limited.

### 2.2. Search Strategy and Data Sources

Due to the highly interdisciplinary nature of this review synthesising clinical epidemiology, algorithmic computer science, and a sociological implementation framework, a traditional, restrictive systematic Boolean search was deemed methodologically inappropriate. Such structured searches frequently fail to capture the intersection of these disparate epistemological domains. Instead, we employed a purposive search strategy across PubMed, Google Scholar, and the Cochrane Library for the literature published between 2000 and 2025. Search terms combined core concepts of medication safety (e.g., “medication errors,” “adverse drug events”) with digital and AI terms (e.g., “predictive analytics,” “machine learning,” “large language models,” “smart pump interoperability”) and implementation science constructs (e.g., “CFIR”). This was supplemented by manually screening the reference lists and national stewardship guidelines (e.g., ACSQHC, ISMP) to identify foundational and emerging texts.

### 2.3. Article Selection

Rather than aiming for exhaustive epidemiological capture, the literature was purposively selected based on its conceptual relevance to the failure modes of the ICU and the architectural components of the proposed Intelligent Safety Stack. Articles were excluded if they focused exclusively on non-critical care populations or lacked relevance to digital, structural, or sociotechnical safety interventions. Ultimately, 41 seminal and highly relevant sources were synthesised to construct the framework.

### 2.4. Data Synthesis

Evidence was synthesised using a best-evidence synthesis approach. Because this review focuses on conceptual framework development across interdisciplinary domains, structured risk-of-bias tools designed for clinical trials were not suitable for evaluating the engineering, algorithmic, and workflow-based literature included. Therefore, “best evidence” prioritised the following: (1) recent (2020–2025) multicentre prospective studies over single-centre retrospective audits; (2) systematic and scoping reviews; and (3) authoritative regulatory frameworks (e.g., ISO standards, ISMP guidelines). Qualitative findings were incorporated to explore the real-world sociotechnical barriers influencing AI adoption, trust, and implementation fidelity.

### 2.5. Limitations

As a narrative review, this methodology inherently lacks the exhaustive reproducibility of a systematic review. Consequently, the claims made regarding the Intelligent Safety Stack represent a hypothesis-generating conceptual model rather than a meta-analytic proof of clinical efficacy. Restriction to English-language publications may also limit generalisability. Furthermore, much of the current evidence evaluates process outcomes rather than patient-centred endpoints (e.g., mortality). Therefore, findings related to AI effectiveness should be interpreted as indicators of error detection and interception rather than confirmed reductions in morbidity and mortality.

Importantly, while the narrative approach allowed for necessary interdisciplinary synthesis, it inherently introduces a risk of selection bias. To mitigate this, our search strategy maintained broad database parameters, utilised extensive reference chaining, and deliberately sought out negative findings—specifically, documented implementation failures, automation bias risks, and instances of technological normalisation of deviance—to ensure the resulting framework remained critically balanced rather than uniformly affirmative.

### 2.6. Use of Generative AI

In accordance with journal policy, the authors declare that Generative AI (Google Gemini 2.0) was used to assist in the conceptual visualisation of figures. All output was critically reviewed and verified by the authors to ensure clinical accuracy and alignment with the manuscript’s findings.

## 3. The Epidemiology of Error: A Methodological Crisis

Establishing the true incidence of MEs in the ICU is complicated by substantial heterogeneity in definitions and detection methodologies. Reported error rates range from 6.8 to 4995 errors per 1000 patient-days, and 1.32% to 31.7% (Table 1), reflecting methodological variance rather than true differences in care quality. This variability exposes a fundamental surveillance gap in which human-dependent reporting systems are incapable of supporting a modern safety culture [7,8].

### 3.1. The Visibility Crisis: Passive Versus Active Detection

**Figure 1:**
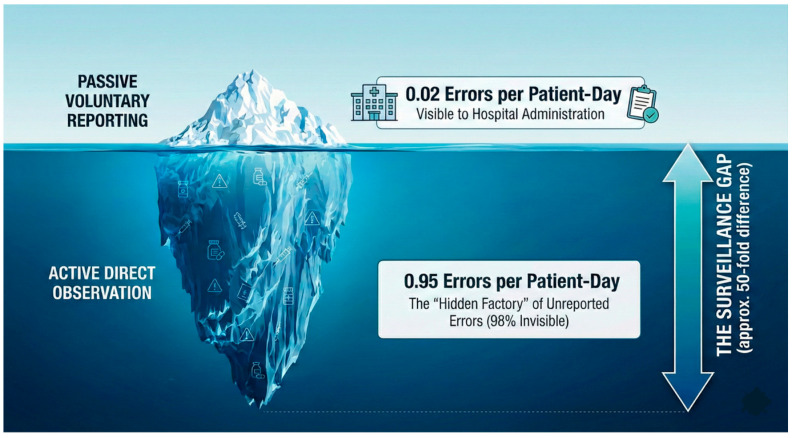
The iceberg of medication errors in the ICU.

A profound discrepancy exists between the incidence captured by passive hospital reporting and active observation. A systematic review of mostly first-world academic tertiary centres concluded that prospective direct observation identified 0.95 MEs per patient-day, whereas passive voluntary reporting captured as few as 0.02—a nearly 50-fold difference, as illustrated in Figure 1 [6]. Westbrook et al. also demonstrated this iceberg phenomenon in a teaching hospital in Australia, showing that direct observation detected 8 to 10 times more errors than hospital reports [13]. This under-reporting is driven by systemic factors, including time constraints, fear of litigation, and a culture where non-harmful errors are normalised rather than documented [15].

### 3.2. The Definition Dilemma

The extreme range of reported error rates is driven primarily by the lack of a consensus definition [9]. While the World Health Organization (WHO) defines an ME broadly as “any preventable event… while the medication is in the control of the healthcare professional,” methodologies apply this inconsistently. Some studies classify minor process variances (e.g., delays) as errors, while others restrict counts to ADEs causing physical harm [16]. Crucially, this lack of semantic consensus prevents AI deployment; without ground truth labels—unambiguous, universally agreed-upon clinical definitions—ML models cannot establish reliable predictive baselines. This necessitates the adoption of common data models, such as the ICURx ontology, to provide the standardised definitions required for the Intelligent Safety Stack [17].

### 3.3. Severity Classification and the Signal-to-Noise Ratio

To operationalise the broad WHO definition, the ICU requires a standardised ontology, such as the NCC MERP Index, to stratify errors by severity (Categories A-I). However, high-fidelity surveillance reveals a signal-to-noise problem: most ICU errors are low-harm events. The multinational SEE study reported that while 46% of patients experienced an error, only 7.5% resulted in clinical harm (Category E+), whereas 51.5% were either intercepted or potential risks [18]. Treating every minor deviation with equal urgency creates data saturation and alert fatigue. Consequently, surveillance must shift to harm-weighted analytics, utilising AI to filter low-grade noise and prioritise genuine risk signals.

### 3.4. The Denominator Problem

Even with standardised definitions, the statistical denominator used to calculate error rates remains imperfect. The convention of reporting errors “per 1000 patient-days” falsely assumes every patient-day carries equal risk, penalising high-acuity units where therapeutic intensity is higher. Researchers advocate normalising rates per 1000 “medication opportunities” to account for exposure volume [19]. While historically labour-intensive to calculate, AI-driven surveillance can now instantaneously aggregate these data points, transitioning metrics from manual sampling to automated, population-level precision.

### 3.5. The Limit of Human Surveillance

Prakash et al. (2025) demonstrate a case for intelligent systems in the ICU by showing that automated trigger tools detect 37.12% of ADEs compared to only 16.54% by manual methods in a tertiary Indian hospital [14]. The automated system excelled at identifying high-risk events through longitudinal data mining—specifically detecting drug-induced hypoglycaemia and opioid-related respiratory depression via reversal agent administration (e.g., dextrose, naloxone)—patterns that manual review frequently missed. However, it should be noted that detecting the administration of a reversal agent signifies that harm has already occurred; this represents the superior detection of systemic failure, rather than the proactive interception required to prevent an ADE.

## 4. Anatomy of Failure: Categorising Error in the ICU

MEs in the ICU do not occur randomly; they cluster around specific stages of the medication-use process, particular drug classes, and vulnerable moments in care delivery. Understanding this anatomy of failure is essential for aligning preventive strategies with the mechanisms through which harm arises, which will be explored in Section 5.

### 4.1. Stage-Based Errors

The literature presents a conflicting picture of error distribution. MEs can occur during any phase: manufacturing, prescribing, transcribing, dispensing, administration, and monitoring, but remain concentrated in the prescribing and administration phases [20]. Retrospective computerised provider order entry (CPOE) data suggest prescription errors account for 63.6% of events. However, this represents a digital surveillance bias; CPOE creates an audit trail for prescribing, while administration errors occur at the unmonitored “sharp end” [21]. Fahimi et al.’s observational data using disguised observation—the gold standard for error detection—identified a higher prevalence of administration errors (9.8%) compared to prescribing faults (6.8%) in the ICU of a Tehran teaching hospital [22]. Unlike prescribing errors, which are frequently detected by pharmacists or decision-support alerts, administration errors lack systemic buffers, occurring at the point of no return where few safety barriers remain.

### 4.2. Procedural vs. Clinical Errors

A distinction must be drawn between procedural errors (deviations from standard workflow) and clinical errors (flaws in decision-making). A Spanish ICU demonstrated that 74.4% of ICU MEs were procedural (versus 25.6% clinical), such as skipping a double-check, mislabelling a line, or programming a pump incorrectly [23]. Procedural errors are failures of implementation fidelity, not knowledge; therefore, they are largely immune to educational interventions. They represent autopilot failures when cognitive bandwidth is saturated in the ICU environment.

### 4.3. Transitions of Care

Transitions of care are periods of vulnerability driven by data fragmentation. Tully et al. identified that, across 58 ICUs in the United States and Netherlands, 47% of patients experience an ME during transitions, with inadvertent omission of chronic medications being the most prevalent [24]. These failures arise not from individual negligence, but from the need to reconstruct medication histories from incomplete, asynchronous data sources (e.g., patient recollection, GP records). While the ACSQHC Medication Safety Standard advocates for formalised “Transition Stewardship”, manual reconciliation is resource-intensive and prone to human oversight [25].

### 4.4. High-Alert Medications and Route Risks

Risk is also disproportionately concentrated among high-alert medications (HAMs), including insulin, anticoagulants, sedatives, and vasoactive agents, which account for 38% of all reported MEs and up to 16% of all medication-related ADEs [26]. The margin for error is non-existent; accidental neuraxial injection (route fatality) remains a fatal risk [27]. Addressing this requires a move away from human vigilance towards fail-proof engineering and technological interventions.

### 4.5. Summary of Classification Strategies

Table 2 synthesises these classifications into an operational framework, linking each error morphology to its specific surveillance challenge and indicating where these vulnerabilities are targeted within the proposed Intelligent Safety Stack in the following section.

## 5. Strategies for Prevention and Mitigation: The Intelligent Safety Stack

Preventing MEs in the ICU involves a transition from isolated, reactive interventions to a coherent, multilayered system designed for intelligent resilience. We define intelligent resilience as the capacity to proactively anticipate, detect, and absorb clinical risks through the fusion of automated AI tools and human oversight, rather than relying on individual human perfection.

**Figure 2:**
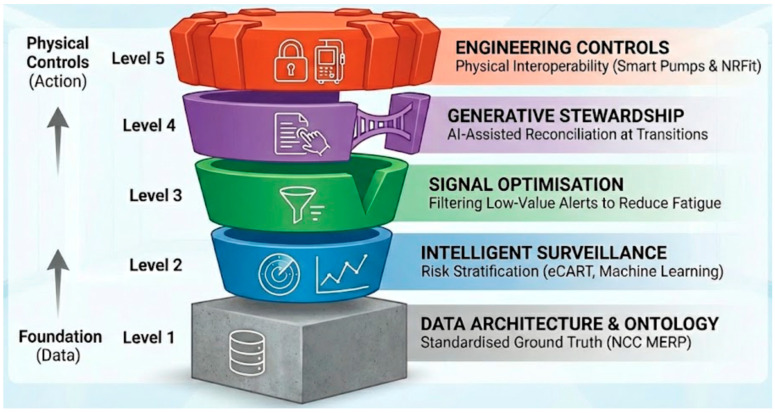
The Intelligent Safety Stack.

Having outlined the objective epidemiology and failure modes in Section 4, this section functions as an interpretive synthesis. We transition from describing the clinical problem to conceptually applying emerging digital and physical interventions, demonstrating how they form the five layers of the Intelligent Safety Stack (Figure 2).

### 5.1. Layer 1: Standardised Data and Error Ontology

The efficacy of AI is strictly bounded by data quality. As established in Section 3, the current lack of a standardised ontology renders much of the EHR data unsuitable for ML training. Therefore, the efficacy of Layer 1 depends on the remediation of data architecture by embedding standardised error taxonomies, such as the NCC MERP Index, directly into the daily workflow of the EHR [18]. Without this foundational standardisation (a core CFIR characteristic), subsequent layers of the stack may struggle to distinguish between benign workflow variations and genuine pre-error signals.

### 5.2. Layer 2: Intelligent Surveillance and Risk Stratification

To address the invisibility of bedside administration errors outlined in Section 4.1, Layer 2 utilises ML to shift from reactive systems to proactive intelligent surveillance. Abdo et al. (2024) utilised an ML-based voting classifier to identify patients at high risk of errors in a French university hospital with a 113% improvement in detection rates compared to standard triage [28]. Furthermore, tools such as the electronic Cardiac Arrest Risk Triage (eCARTv5) model integrate gradient-boosted ML to patient observations [9]. In a medication safety context, these models serve as a secondary defence: if an erroneous dose bypasses upstream checks, the resulting physiological instability is detected as a high-risk deviation from the patient’s baseline—identifying subtle multivariate trends before single-parameter telemetry alarms are triggered—prompting earlier bedside assessment. While this represents a significant advancement in signal detection, improved algorithmic sensitivity does not inherently guarantee harm prevention. The translation from a digital alert to a successful clinical interception remains entirely dependent on the timeliness and efficacy of the human clinician’s response.

Implementation barrier: Translation is further obstructed by algorithmic opacity. Unlike current systems where a clinician can trace the logic of a score, deep learning models process data through hidden layers, generating predictions without revealing the specific features that drove them. Tun et al. (2025) identified that this lack of transparency is a primary driver of implementation failure; clinicians actively reject alerts that lack transparent reasoning, viewing them as clinically indefensible [29]. Consequently, Layer 2 requires explainable AI (XAI) dashboards that provide feature attribution. Trust in the ICU is contextually bounded; even a mathematically superior algorithm will be ignored if it fails to provide feature attribution explaining why the patient was flagged. Furthermore, success depends on their integration into existing workflows—even robust models are clinically inert without governance protocols empowering staff to act on high-risk signals [30].

### 5.3. Layer 3: Intelligent Clinical Decision Support and Signal Optimisation

While Layer 2 focuses on generating new safety signals, Layer 3 addresses the suppression of non-actionable alerts. With 90% of alerts being overridden and 56.5% of clinicians reporting being overwhelmed, decision quality is compromised [5,31]. A 2024 review by Graafsma et al. found that ML models trained on historical acceptance patterns could accurately predict and suppress low-value warnings [32]. By filtering noise, these mechanisms preserve cognitive bandwidth for high-stakes decisions [32].

Implementation barrier: A validation gap exists as most evidence is simulation-based. Therefore, widespread adoption requires prospective non-inferiority trials to demonstrate that AI-filtered alert streams are statistically as safe as the unfiltered output of current systems, avoiding the crucial error of a genuine alert that has been incorrectly silenced.

### 5.4. Layer 4: Generative Stewardship at Transitions of Care

Layer 4 addresses the 47% error rate at transitions of care [24]. We introduce the concept of generative stewardship: the application of generative AI to augment clinician cognition by synthesising fragmented, unstructured patient records into coherent reconciliation lists, while maintaining a human-in-the-loop for final adjudication. Kabir et al. (2025) demonstrated that AI-assisted admission workflows reduced unintentional medication discrepancies (incidence rate ratio 0.82) and severe potential ADEs (odds ratio 0.69), while reducing completion time by 24% in a tertiary academic hospital in Bangladesh [33]. Similarly, Ong et al. (2025) found that a workflow involving a pharmacist–LLM collaboration achieved a diagnostic accuracy of 61%, significantly outperforming pharmacists working alone (46%) [34].

Implementation barrier: The risk of hallucination prevents autonomous deployment [35]. Ong et al. noted that, while the LLMs excelled at identifying interactions, its accuracy declined in dosing appropriateness (52%) compared to human experts [34]. Therefore, Layer 4 requires a “human-in-the-loop” governance structure: AI drafts the reconciliation list, but the pharmacist acts as the final adjudicator.

### 5.5. Layer 5: Engineering Controls

The final layer addresses the physical sharp end of administration. Digital alerts are often invisible during this phase, necessitating engineering controls. The primary intervention is bi-directional smart pump interoperability. Historically, infusion safety relied on manual programming, a workflow susceptible to keystroke errors and transcription faults. The contemporary standard, aligned with Institute for Safe Medication Practices (ISMP) Best Practice 8, requires the EHR to directly program the infusion device [36]. Borrelli et al. (2025) demonstrated that this integration reduced administration errors by 15.4% to 54.8% in multi-hospital settings, including the ICU [10].

Complementing this is the prevention of wrong-route errors through physical incompatibility. As mandated by NHS England (2024), ISO 80369-6 (NRFit™) standard uses a unique collar design that is mechanically incompatible with standard Luer-locks, physically preventing catastrophic wrong-route errors regardless of clinician vigilance [11,37,38].

Implementation barrier: Efficacy is compromised by workflow rigidity and the normalisation of deviance. If interoperability workflows are slower than manual programming, staff may bypass safety software to maintain throughput.

These failures are not instances of arbitrary non-compliance, but predictable human responses to system design flaws. Specifically, when evaluating the CFIR construct of Relative Advantage, if bi-directional interoperability workflows are perceived by bedside nurses as slower or more cognitively burdensome than traditional manual programming, staff will predictably bypass the safety software to maintain critical care throughput. This empirical reality demonstrates that technological superiority alone does not guarantee implementation fidelity. To succeed, these systems must demonstrate high Adaptability—the CFIR construct defining a tool’s ability to be tailored to local, fast-paced ICU workflows. Practically, this means aligning intelligent pump library soft-limits with actual, unit-specific prescribing habits to ensure the safest workflow is simultaneously the most efficient, thereby systematically engineering out the incentive for dangerous workarounds [10].

## 6. Discussion: The Sociotechnical Pivot

The evidence indicates that the landscape of ICU medication safety is undergoing a fundamental paradigm shift from human vigilance-based prevention to intelligent resilience. Digitisation alone has failed to deliver the anticipated gains and has, in some cases, introduced new hazards through cognitive overload and alert fatigue. The future of safety lies not in further digitisation, but in sociotechnical integration.

### 6.1. The Paradox of Detection and Operationalising Rescue Rates

A key implication of high-sensitivity intelligent surveillance (Layer 2) is the paradoxical increase in detected errors. Improved visibility reveals latent risk rather than deteriorating performance. Consequently, traditional metrics centred on raw error counts become misleading during implementation. Performance assessment must evolve to include measures such as interception and rescue rates, making evaluation of intelligent resilience tools possible.

To transition this concept from a rhetorical construct to an operational metric, we define the rescue rate as the proportion of high-risk medication variances successfully intercepted before reaching the patient. It can be formally calculated as:

$$Rescue\;rate=\frac{Validated\;interceptions\;(NCC\;MERP\;Categories\;B-D)}{Total\;detected\;errors\;(NCC\;MERP\;Categories\;B-I)}\times 100$$

We bind the numerator to Categories B through D to capture true interceptions: instances where an error occurred but was buffered before causing harm. This excludes environmental hazards where no actual error occurred (Category A) and instances where systemic failure resulted in patient harm (Categories E–I).

For this metric to be scientifically robust, counterfactual harm—the theoretical harm averted—must be inferred using these standardised taxonomies. For example, if an AI surveillance tool flags a narrow-therapeutic-index ME that is subsequently intercepted (Category C), the counterfactual harm is estimated based on the historical severity of that specific medication class, reaching Category E or higher.

To transition the rescue rate from a conceptual proposal to a systems-level metric, an operational framework is required. First, we advocate for the adjudication of NCC MERP categories to be a dual independent review process (e.g., a clinical pharmacist and an intensivist), utilising a multidisciplinary consensus panel to resolve discrepancies and ensure inter-rater reliability. Second, counterfactual harm should be calculated via probabilistic matching with historical, institution-specific adverse event databases, stratifying risk by medication class and patient acuity. Third, to mitigate detection-intensity bias where the deployment of high-sensitivity Layer 2 surveillance artificially inflates the number of recorded rescues simply by detecting more latent risks, rates should be normalised per 1000 automated medication opportunities rather than raw patient-days [19]. Finally, because rescue opportunities are inherently tied to therapeutic intensity, raw metrics cannot be directly compared across different hospitals. Benchmarking requires risk-adjustment models that account for case-mix differences, ensuring that tertiary ICUs managing highly complex, polypharmacy patients are not disproportionately penalised when comparing safety performance.

### 6.2. System Interactions and Cross-Layer Failure Propagation

While the Intelligent Safety Stack is presented as a layered architecture, it is crucial to recognise that these layers operate within a dynamic, non-linear clinical environment. This produces a risk of cross-layer failure propagation, where improvements or aggressive tuning in one layer may inadvertently degrade the performance of another.

**Figure 3:**
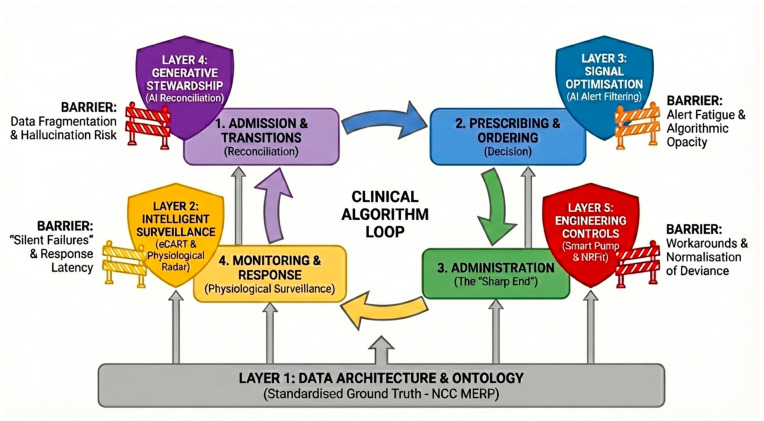
The protected workflow: mapping the Intelligent Safety Stack to clinical reality. Note: This model is a conceptual, non-sequential architecture rather than a linear maturity-stage model. In clinical practice, these layers operate simultaneously and are characterised by dynamic, bidirectional feedback loops and complex system interactions.

For example, if Layer 3 (Signal Optimisation) is calibrated too aggressively to suppress low-value notifications and reduce alert fatigue, it risks creating a silent failure by inadvertently filtering out a rare but critical physiological deterioration signal generated by Layer 2 (Intelligent Surveillance). Similarly, a highly efficient Generative AI output in Layer 4 (Generative Stewardship) might produce a flawlessly formatted but clinically hallucinated reconciliation list. If this encourages automation bias, the clinician may bypass standard cognitive checks, allowing the erroneous dose to pass directly into Layer 5, where an interoperable smart pump will flawlessly execute the incorrect order. Therefore, the sociotechnical framing of the Stack demands continuous monitoring of feedback loops and system interactions (as partially visualised in Figure 3), ensuring that the mitigation of one hazard does not become the vector for another.

### 6.3. Workforce Transformation

The integration of generative stewardship (Layer 4) also reshapes professional roles. By automating low-value, repetitive tasks such as manual reconciliation and transcription, generative stewardship frees pharmacists, nurses, and physicians to focus on complex decision-making and individualised therapy. Belcher et al. (2023) demonstrated that tele-critical care pharmacists overseeing multiple community hospital ICUs in the United States generated a 4.5:1 return on investment (ROI) and $1.6 million in cost avoidance [39]. Although Belcher’s study utilised remote telehealth rather than AI, it supports the economic principle that technology-enabled clinical oversight generates significant ROI.

But we must acknowledge that an over-reliance on generative stewardship can introduce epistemic risk and clinician de-skilling. If an AI-drafted reconciliation list appears flawless, clinicians may subconsciously bypass their own cognitive checks, assuming the machine has already resolved any discrepancies. This introduces the dual threats of automation bias and responsibility diffusion.

If junior medical staff routinely defer to generative algorithms for pharmacological reconciliations, they may lose the foundational cognitive skill and knowledge to perform these tasks independently when systems fail or present ambiguous data. Consequently, Layer 4 necessitates a strict human-in-the-loop governance structure: the AI is restricted to drafting the reconciliation list, while the clinical pharmacist or attending physician acts as the mandatory, active adjudicator.

### 6.4. The Digital Determinants of Safety

The transition to the Intelligent Safety Stack introduces an equity risk. The required technologies (predictive servers, interoperable pumps) demand substantial capital. This introduces concern that technological stratification may create a digital divide in care quality, where under-resourced ICUs remain dependent on fallible human vigilance while wealthy institutions leverage algorithmic safety nets. Future implementation frameworks must consider scalability, equity, and adaptability to avoid widening global disparities in critical care outcomes.

To mitigate this systemic disparity, implementation frameworks must define a minimum viable safety architecture tailored for resource-constrained environments. This architecture prioritises low-cost, high-impact structural and procedural safeguards over capital-intensive digital tools. For example, standardising data and error ontology (Layer 1) requires governance consensus rather than heavy financial investment. Similarly, mandating physical forcing functions, such as NRFit™ connectors to prevent wrong-route errors (Layer 5), provides absolute mechanical safety at a fraction of the cost of ML integration. Furthermore, strategic workforce design such as the inclusion of clinical pharmacists during ICU ward rounds can replicate many of the interception benefits of generative stewardship without the associated IT (information technology) overhead [1]. Future research and national safety guidelines must explicitly outline these scalable, equitable pathways to ensure that intelligent resilience does not become an exclusive commodity.

### 6.5. Limitations of the Evidence and Future Research Agenda

The literature is constrained by two fundamental gaps that define the research agenda for 2026 and beyond. First, a validation crisis exists; most studies measure algorithmic accuracy (retrospective efficacy) rather than patient outcomes (prospective effectiveness), creating an outcome gap. Without prospective validation, the true clinical utility of tools like intelligent surveillance remains theoretical. A primary vulnerability is dataset shift—the phenomenon where an algorithm trained on the specific demographic, pharmacological, and workflow patterns of one ICU fails to generalise to another. These tools are also highly susceptible to post-deployment performance decay (model drift). As clinical guidelines evolve and patient demographics fluctuate, a ML model will progressively lose its predictive calibration. Consequently, robust external validation and a commitment to continuous, site-specific algorithm recalibration are essential. Without rigorous post-market surveillance, the uncritical deployment of AI risks replacing isolated human error with scaled algorithmic failure.

Second, implementation fidelity is consistently underreported; without data on user adherence, it is impossible to distinguish between theory failure (flawed algorithm) and implementation failure (flawed rollout). Future research should prioritise hybrid-effectiveness designs to evaluate both tool performance and deployment strategy.

### 6.6. Implementation Priorities and Practice Recommendations

To operationalise the findings of this review, we categorise interventions into a tiered roadmap for ICU leaders. The resulting hierarchy, outlined in Table 3, distinguishes between fundamental policy standards targeted for early adoption and emerging technologies that require infrastructure maturity. Complementing this strategic list, Figure 3 visualises the tactical integration of these layers into the clinical workflow. This “Protected Workflow” highlights where sociotechnical barriers threaten implementation fidelity.

### 6.7. Governance and Clinical Liability

The integration of the Intelligent Safety Stack introduces profound medicolegal challenges that require regulatory attention. As Minssen et al. (2020) demonstrate, current regulatory frameworks governing Software as a Medical Device (SaMD) are fundamentally misaligned with the adaptive, continuously learning nature of modern ML models [40]. This regulatory lag exacerbates what Habli et al. (2020) describe as the “problem of many hands” in healthcare AI: when an algorithmic error occurs such as Layer 3 incorrectly suppressing a critical alert, accountability is dangerously fragmented among software developers, hospital IT infrastructure, and bedside clinicians [41]. Their findings suggest that relying on traditional models of individual clinician blame is entirely insufficient for systems-level algorithmic failures.

At the bedside, this fragmentation creates a liability trap for intensive care physicians. Price et al. (2019) highlight that the legal “standard of care” is currently unequipped to handle AI-augmented decision-making [42]. If a clinician follows an AI-generated reconciliation list (Layer 4) that contains a hallucinated error, or conversely, overrides an AI surveillance alert (Layer 2) that was actually correct, establishing negligence becomes highly complex. Price and colleagues argue that, without explicit legal safe harbours, clinicians are placed in an untenable position where adhering to or defying an algorithm carries equal, unpredictable legal risk. Therefore, before AI recommendations are permitted to heavily influence critical care, facilities are urged to establish institutional governance that defines the parameters of “human-in-the-loop” accountability and protects clinicians from the opaque liabilities of algorithmic systems.

## 7. Conclusions

The persistence of medication errors in the ICU, with rates as high as 0.95 per patient-day, demonstrates that safety strategies based on human vigilance and voluntary reporting have inherent limitations. The surveillance gap, where 98% of errors remain invisible to retrospective evaluation, confirms that current reporting protocols are insufficient. We argue that this persistence is not a failure of individual character, but a failure of system design.

Sustainable safety requires operationalising the Intelligent Safety Stack as a cohesive, sociotechnical defence system. This begins with a standardised data ontology (Layer 1) to enable reliable analytics, followed by intelligent surveillance (Layer 2) and signal optimisation (Layer 3) to proactively identify and monitor high-risk patients and suppress low-value noise. At transitions of care, generative stewardship (Layer 4) can automate reconciliation to bridge data fragmentation, while engineering controls (Layer 5) provide the final physical hard-stop against administration errors at the bedside.

Ultimately, the metric for safety requires evolution. To achieve the WHO’s *Medication Without Harm* mandate, ICUs are urged to transition from raw error counting to measuring rescue rates—the successful interception of risk through intelligent, human-in-the-loop collaboration. The future of critical care safety lies not in expecting clinicians to be perfect, nor in surrendering oversight to opaque algorithms, but in designing integrated systems that make it difficult to fail.

## Figures and Tables

**Table 1:** The surveillance gap: impact of methodology on reported error rates.

Study *	Study Design (Methodology)	Reported Incidence/Prevalence	Key Limitation/Insight
Wilmer et al. (2010) [6]	Systematic Review (Direct observation vs. Voluntary reporting)	0.95 MEs (Observation) vs. 0.02 MEs per patient-day (Reporting)	Defines the surveillance gap: passive reporting misses 98% of errors; proves voluntary systems are functionally blind.
Westbrook et al. (2010) [13]	Prospective, observational (Direct Observation: IV administration focus)	41% of IV doses had ≥1 error; Found 8–10× more errors than hospital reports.	Highlights the specific vulnerability of the administration phase, often invisible to retrospective chart audits.
Kwiecień-Jaguś et al. (2025) [7]	Systematic Review (*n* = 13 studies)	ICU: 1.32–31.7%	Extreme heterogeneity in reported error rates is driven by inconsistent definitions and detection methods (prospective observation vs. retrospective records).
Puxty et al. (2025) [8]	Scoping Review (48 global studies)	Range: 6.8 to 4995 MEs per 1000 patient-days.	Extreme variance highlights the lack of a standardised ground truth definition across the critical care community.
Prakash et al. (2025) [14]	Prospective, single-centre, observational Automated Surveillance: EHR Trigger Tools vs. Manual	37.12% ADE detection (Automated) vs. 16.54% ADE detection (Manual)	Confirms that automated mining of longitudinal data (e.g., reversal agents) is significantly more sensitive than human vigilance.

* Listed are: first author, year of publication, and reference number. Note: MEs: Medication Errors; IV: Intravenous; ADE: Adverse Drug Event; EHR: Electronic Health Record.

**Table 2:** The evolving taxonomy of ICU medication errors and digital remediation strategies.

Classification Type	Definition & Key Statistic	The 2025 Challenge	Proposed Stack Intervention
Stage-Based	Phase of Use (Prescribing vs. Administration phase).	Surveillance Bias: CPOE makes prescribing errors highly visible, while bedside administration slips remain invisible to retrospective digital audits.	Row 1 (Stage-Based): Layer 2 & Layer 5 (See Section 5.2 and Section 5.5)
Procedural vs. Clinical	Slips vs. Mistakes. 74.4% of ICU errors are procedural (e.g., missed check, wrong time) rather than clinical judgement errors.	Implementation Fidelity: Procedural errors are failures of workflow, not knowledge. Education has limited impact on these autopilot failures.	Row 2 (Procedural): Layer 5 (See Section 5.5: Engineering Controls)
Route-Based	Catastrophic risk of wrong-route administration.	Physical Compatibility: Human visual checks are unreliable under stress; reliance on vigilance for line connection is unsafe.	Row 3 (Route-Based): Layer 5 (See Section 5.5: Engineering Controls)
High-Alert Medications	HAMs (Insulin, Heparin, Vasopressors) account for 38% of errors and 16% of all ADEs.	Zero Tolerance: Narrow therapeutic indices mean that even minor deviations (e.g., decimal point errors) result in rapid patient deterioration.	Row 4 (High-Alert): Layer 3 & Layer 5 (See Section 5.3 and Section 5.5)

Note: CPOE: Computerised Provider Order Entry; HAMs: High-Alert Medications; ADEs: Adverse Drug Events.

**Table 3:** The 2025 Intelligent Safety Stack: priorities and barriers.

Layer/Component	Intervention	Evidence & Clinical Impact (Study Design)	Primary Barrier (CFIR Construct)	2025 Implementation Action
Layer 1: Data Architecture	Standardised Error Ontology	Prerequisite for AI: Without ground truth definitions, predictive models fail to distinguish workflow variance from error.	Structural Characteristics: Lack of semantic interoperability prevents cross-site validation.	Embed NCC MERP definitions directly into EHR workflows to standardise error data capture.
Layer 2: Surveillance	Intelligent Surveillance	Proactive Detection: ML-based voting classifiers improved error detection by 113% compared to standard triage. (Retrospective single-centre validation; Process outcome)	Culture (Trust Deficit): Algorithmic opacity drives algorithm aversion; clinicians reject alerts lacking explainability.	Implement XAI dashboards that display feature attribution (why the patient was flagged).
Layer 3: Optimisation	Intelligent Signal Filtering	Cognitive Protection: ML models can predict and suppress low-value alerts that generate 90% override rates. (Scoping review of simulation-based models; Process outcome)	Evidence Strength: Validation crisis; current evidence is largely simulation-based, risking silent failures (false negatives).	Conduct prospective non-inferiority trials to prove filtered streams are as safe as unfiltered baselines.
Layer 4: Stewardship	Generative AI Reconciliation	Workflow Augmentation: LLM-assisted reconciliation reduced discrepancies (IRR 0.82) and saved ~15 min per admission. (Prospective before-and-after study; Clinical & Efficiency outcome)	Compatibility (Risk): Probabilistic hallucination in dosing appropriateness (52% accuracy) requires supervision.	Mandate clinician governance: AI drafts the list, Pharmacist acts as final adjudicator.
Layer 5: Engineering	Smart Pump Interoperability	Closed-Loop Safety: Bi-directional integration reduces administration errors by 15.4% to 54.8%. (Systematic literature review; Clinical outcome)	Adaptability: Workflow rigidity encourages normalisation of deviance (workarounds) if pump programming is slow.	Align pump library soft limits with actual prescribing habits to ensure the safe workflow is also the efficient workflow.
	NRFit™ (ISO 80369-6) [38]	Physical Forcing Function: Mechanically prevents catastrophic wrong-route neuraxial errors.	Available Resources: Requires complete stock turnover of spinal/epidural trays.	Audit all procedural trays; mandate total transition to route-specific connectors.

Note: NCC MERP: National Coordinating Council for Medication Error Reporting and Prevention; ML: Machine Learning; XAI: Explainable AI; LLM: Large Language Model; IRR: Incidence Rate Ratio; CFIR: Consolidated Framework for Implementation Research.

## Data Availability

No new data were created or analysed in this study.
